# Improvements in Immune Function and Activation with 48-Week Darunavir/Ritonavir-Based Therapy: GRACE Substudy

**DOI:** 10.1155/2013/358294

**Published:** 2013-12-12

**Authors:** Christos Tsoukas, Louise Gilbert, Trevor Lewis, George Hatzakis, Ron Falcon, Joseph Mrus

**Affiliations:** ^1^Department of Microbiology and Immunology, McGill University Health Centre, Room A5-140, 1650 Cedar Avenue, Montreal, QC, Canada H3G 1A4; ^2^Montreal General Hospital, Flowcytometry Laboratory, 1650 Cedar Avenue, Room C10-166, Montreal, QC, Canada H3G 1A4; ^3^LAC+USC Medical Center, 1200 N State Street, Room IPT-C4E100, Los Angeles, CA 90033, USA; ^4^Janssen Services, LLC, Clinical Affairs, 1125 Trenton-Harbourton Road, Titusville, NJ 08560, USA

## Abstract

*Objective*. During the course of HIV infection, progressive immune deficiency occurs. The aim of this prospective substudy was to evaluate the recovery of functional immunity in a subset of patients from the GRACE (Gender, Race, And Clinical Experience) study treated with a DRV/r-based regimen. *Methods*. The recovery of functional immunity with a darunavir/ritonavir-based regimen was assessed in a subset of treatment-experienced, HIV-1 infected patients from the GRACE study. *Results*. 19/32 patients (59%) enrolled in the substudy were virologically suppressed (<50 copies/mL). In these patients, median (range) CD4+ cell count increased from 222 (2, 398) cells/mm^3^ at baseline to 398 (119, 812) cells/mm^3^ at Week 48. CD8+% decreased significantly from baseline to Week 48 (*P* = .03). Proliferation of CD4+ lymphocytes in response to CD3+/CD28+, phytohemagglutinin, and pokeweed was significantly increased (*P* < .01) by Week 12. Proliferation in response to *Candida* and tetanus was significantly increased by Week 48 (*P* < .01 and *P* = .014, resp.). Staphylococcal enterotoxin B-stimulated tumor necrosis factor-alpha and interleukin-2 in CD4+ cells was significantly increased by Week 12 (*P* = .046) and Week 48 (*P* < .01), respectively. *Conclusions*. Darunavir/ritonavir-based therapy demonstrated improvements in CD4+ cell recovery and association with progressive functional immune recovery over 48 weeks. This trial is registered with NCT00381303.

## 1. Introduction 

During the course of HIV-1 infection, multifactorial T-lymphocyte (T-cell)-mediated mechanisms contribute to the progressive loss of host immune function [1–5]. In infected individuals, immune dysregulation occurs early and is characterized by a decrease in CD4+ cell count, a concurrent rise in CD8+ cells, a progressive decline in the CD4+/CD8+ ratio, and defective thymocyte proliferation [6]. During late-stage disease, loss of T-cell homeostasis also occurs [7, 8].

T cells are chronically activated throughout the course of HIV infection, as indicated by an increase in the expression of the antigens Ki67, CD38, and human leukocyte antigen (HLA)-DR, with CD38 recognized as the most reliable marker of immune activation [1–3, 5, 9]. Immune activation provides the virus with a steady pool of target cells and has been linked with increased polyclonal T-cell proliferation and turnover, as well as increases in the apoptotic marker CD95 [10–13] and activation-induced cell death [12, 14–16].

Concomitant with the decline of CD4 cells in the peripheral blood, the frequency of the CD4+ CD28 null subset increases with disease progression and eventual progression to AIDS [10]. The presence of CD28 on T cells is critically important for the generation of T-cell responses. Interaction of this costimulatory molecule with its ligands increases the expression of antiapoptotic proteins and improves interleukin (IL)-2 production. The increase in circulation of T cells with a CD4+ CD28 null phenotype is consistent with a process known as replicative senescence [11, 13, 17–19]. T cells that lack CD28 surface expression are nonanergic, oligoclonally expanded, and terminally differentiated, with limited replicative capacity and increased sensitivity to apoptosis [20, 21]. These alterations in phenotype are accompanied by cytokine changes consistent with a chronic proinflammatory state. The failure to produce adequate amounts of IL-2 leads to a marked functional impairment of cellular and humoral immunity. Reduced IL-2 expression has been associated with a shift from Th1 cytokine responses to Th2 cytokine responses. It has been postulated that the Th1/Th2 imbalance may result in cellular immunity deficiency [4].

The dysregulation of cytokine secretion during the course of HIV infection has been examined using a wide range of methods, including enzyme-linked immunosorbent assay, reverse transcriptase-polymerase chain reaction, T-cell cloning, and, more recently, flow cytometric intracellular cytokine detection [4]. Of these methods, only flow cytometry allows quantitative and qualitative determination of cytokine expression patterns in individual T cells [4].

Successful antiretroviral (ARV) therapy results in improvements in circulating CD4+ T-cell levels with decreases in CD8+ counts and declines in immune activation and CD95 expression [1–5, 7, 9, 22–26]. Limited data on replicative senescence suggest a lack of significant improvement in the short term, despite adequate HIV suppression [10]. Some studies have suggested that protease inhibitor- (PI-) based regimens may have a greater effect on CD4+ count recovery than nonnucleoside reverse transcriptase inhibitor-based regimens [27, 28]. Some of the previous studies on immune recovery with ARV therapy have been cross-sectional in design, conducted primarily in white males, and focused on absolute CD4+ count or CD4+% recovery. Furthermore, most of these studies did not comprehensively assess the functional aspects of immune recovery [29]. Of the studies that did evaluate the influence of ARV therapy on functional immune parameters, most were drug class specific [30–32].

GRACE (Gender, Race, And Clinical Experience) is the largest ARV trial to focus on women with HIV-1 in North America and was designed to assess sex-based and race-based differences in efficacy, safety, and tolerability of the PI darunavir with low-dose ritonavir (DRV/r) plus an optimized background regimen over 48 weeks in a diverse, treatment-experienced patient population [33]. The aim of this prospective substudy was to quantitatively and qualitatively evaluate the recovery of functional immunity (T-cell function) with a DRV/r-based regimen in a subset of patients from the GRACE study.

## 2. Materials and Methods

### 2.1. Study Design and Patients

GRACE was a multicenter, open-label, single-arm, Phase IIIb study conducted at 65 sites across USA, Puerto Rico, and Canada that enrolled 429 treatment-experienced patients (viral load ≥ 1000 HIV-1 RNA copies/mL) aged at least 18 years with documented HIV-1 infection [33]. Full inclusion and exclusion criteria and the study flow diagram have been reported [33]. Patients received DRV/r 600/100 mg twice daily plus an investigator-selected background regimen that could include etravirine. Patients at participating GRACE study sites were eligible for the prospective immunology substudy, which aimed to enroll up to 100 subjects. A group of healthy, HIV seronegative volunteers were included for comparison.

### 2.2. Ethics Statement

The research has complied with all federal guidelines and institutional policies. All subjects were required to sign a separate, Independent Ethics Committee/Institutional Review Board (IRB) informed consent form specific to this substudy. The IRBs were Quorom Review Inc., Seattle, WA, Office of Human Research Ethics—UNC IRB, Chapel Hill, NC, and AIDS Research Consortium of Atlanta, Atlanta, GA. Each IRB approved of this study. The study was conducted in accordance with the principles of the Declaration of Helsinki and followed Good Clinical Practice guidelines.

### 2.3. Study Evaluations

Viral suppression in this analysis was defined as achieving HIV-1 RNA less than 50 copies/mL at Week 48. Immune function and phenotype were determined by flow cytometry at baseline and Weeks 12 and 48 in virologically suppressed and nonsuppressed patients. Changes in immune phenotype were determined from subsets of CD4+ and CD8+ T cells, with immune activation defined as increased expression of T-cell CD38 and HLA-DR surface markers, and immune replicative senescence by increased frequency of circulating CD28 null T cells. Changes in immune function were assessed by lymphocyte proliferation in response to *Candida* and tetanus (recall antigens), phytohemagglutinin (PHA) and pokeweed (mitogenic plant lectins), and CD3+/CD28+ and by intracellular cytokine expression of IL-2, interferon-gamma (IFN-*γ*), and tumor necrosis factor-alpha (TNF-*α*) in response to staphylococcal enterotoxin B.

### 2.4. Immunophenotyping

A whole blood lysing technique was used and subpopulations were assessed according to a standard protocol for 3-color and 4-color immunofluorescence flow cytometry using fluorochrome conjugated monoclonal antibodies and a fluorescence activated cell sorter. A panel of monoclonal antibodies was used to delineate total CD3+, total CD4+ and CD8+ T cells, CD4+/CD8+ ratio, activated T cells (CD38+/HLA-DR+), immune replicative senescence (CD4+/CD28+ or CD8+/CD28+), and apoptotic cells (CD95+).

### 2.5. Proliferation Assays

The Vybrant CFDA SE Cell Tracer Kit (Molecular Probes, Inc.) was used. Lymphocytes were labeled with carboxyfluorescein diacetate and succinimidyl ester CFDA SE and then incubated at 37°C with 5% carbon dioxide for 7 days. The label is inherited by daughter cells after division. Labeled lymphocytes with carboxyfluorescein diacetate and succinimidyl ester were detected by flow cytometry using CD45 markers for gating strategy. This method quantified mitogenic and recall antigen responses. The reagents used were *Candida*, tetanus, CD3+/CD28+, PHA, and pokeweed.

### 2.6. Intracellular Cytokines

Flow cytometric intracellular cytokine detection of Th1 cytokines including IFN-*γ*, IL-2, and TNF-*α* was assessed using the BD Cytofix/Cytoperm Plus Fixation/Permeabilization (GolgiPlug protein transport inhibitor; BD Biosciences). Peripheral blood mononuclear cells were stimulated with staphylococcal enterotoxin B (Sigma) and a CEF control peptide pool (CMV, EBV, Influenza virus; NIH reagent 9808). This stimulation step was followed by the fixation and permeabilization of the cells. Then, surface and intracellular staining antibodies were added in a single staining step (Anti-Hu-IFN-*γ*/CD69/CD4/CD3, Anti-Hu-IL2/CD69/CD4/CD3, Anti-Hu-TNF-*α*/CD69/CD4/CD3; BD Biosciences). Cells were acquired and analyzed by flow cytometry.

### 2.7. Statistical Analysis

Immune recovery was evaluated in continuous and categorical outcome analyses. Wilcoxon rank-sum tests were used to assess immune parameter changes from baseline.

## 3. Results

### 3.1. Patient Population and Baseline Characteristics

A total of 32 patients with HIV-1 were enrolled in the substudy; 25 (78%) patients had Week 48 data and 19 (59%) were virologically suppressed at Week 48. The normal comparator group consisted of 34 healthy, HIV-seronegative individuals, 17 (50%) of whom were women and 25 (74%) of whom were white. Patient demographics and baseline characteristics for total and virologically suppressed patients are shown in Table 1.

### 3.2. Immune Phenotype

**Figure 1:**
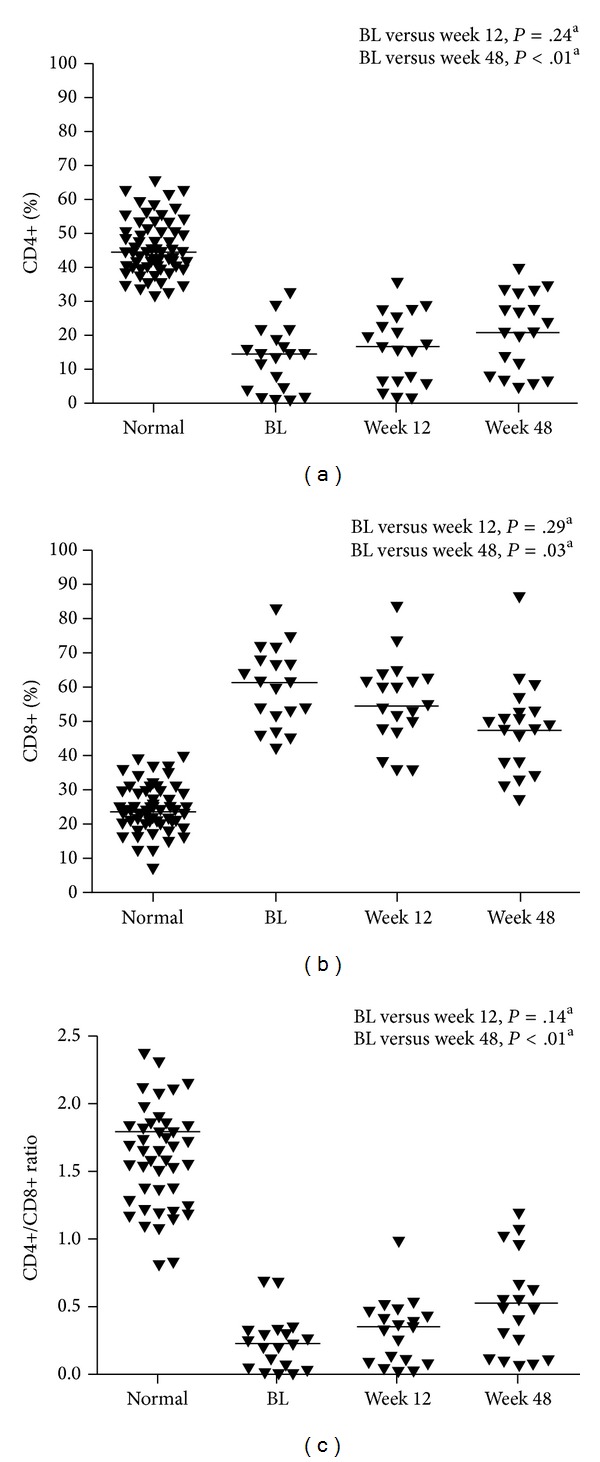
(a) Percent CD4+, (b) percent CD8+, and (c) CD4+/CD8+ ratio in virologically suppressed patients. Line represents median; ^a^Wilcoxon rank-sum test; BL: baseline.

The median (range) CD4+ count at baseline for the total patient population was 191 (2, 463) and the median (range) CD4+ count in virologically suppressed patients at baseline was 222 (2, 398) cells/mm^3^ (Table 1). At Week 48, the median (range) CD4+ count for the total population and virologically suppressed patients was 337 (98, 812) and 398 (119, 812) cells/mm^3^, respectively. In virologically suppressed patients, the CD4+% increased significantly from baseline to Week 48 (*P* < .03; Figure 1). The median (range) CD8+ count at baseline for the total patient population was 912.5 (288, 3131) cells/mm^3^, while the median (range) CD8+ count in virologically suppressed patients at baseline was 1037 (288, 3131) cells/mm^3^. The CD8+% decreased significantly from baseline to Week 48 (*P* < .01) in virologically suppressed patients (Figure 1). The median (range) CD4+/CD8+ ratio at baseline was 0.22 (0.01, 0.70) in all patients and 0.22 (0.01, 0.70) in virologically suppressed patients. The CD4+/CD8+ ratio at Weeks 12 and 48 is displayed in Figure 1 and Table 2. The CD4+/CD8+ ratio increased significantly from baseline to Week 48 (*P* < .01) in suppressed patients. The percentage of CD4+ and CD8+ cells at Weeks 12 and 48 in suppressed patients is shown over 48 weeks in Figure 1. The percentage of CD4+ cells significantly increased (*P* < .01) and CD8+ cells significantly decreased (*P* = .03) from baseline to Week 48.

**Figure 2:**
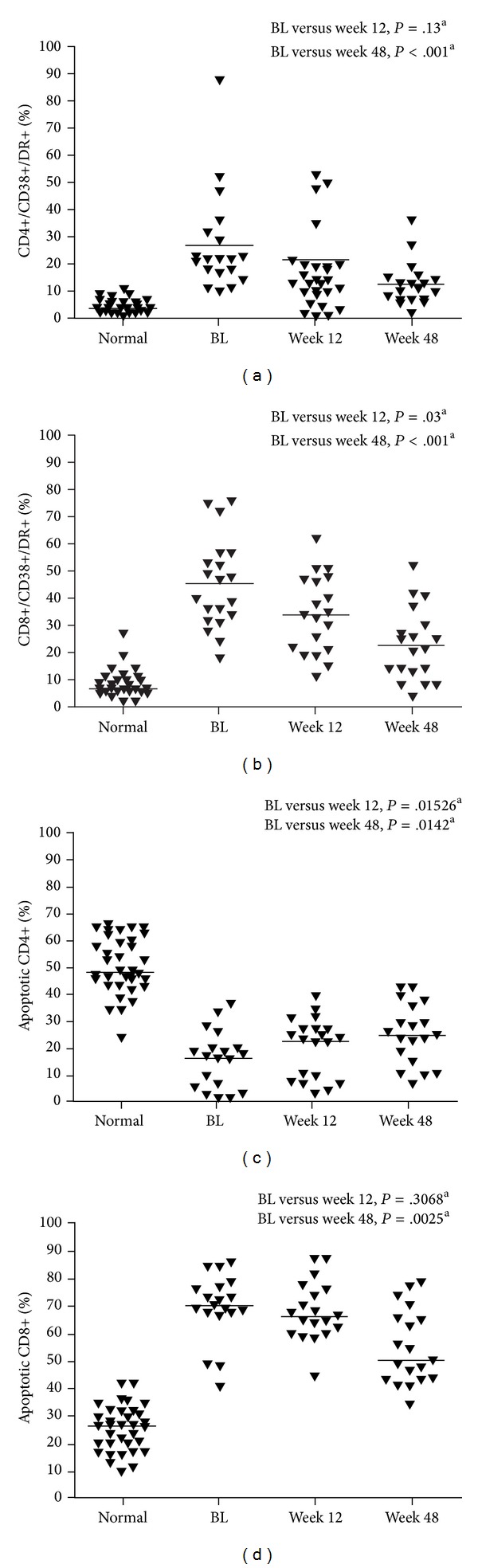
(a) CD4+/CD38+/DR+, (b) CD8+/CD38+/DR+, percentage of apoptotic T cells, (c) CD4+/CD95+, (d) CD8+/CD95+. Line represents median; ^a^Wilcoxon rank-sum test; BL: baseline.

Improvements in immune activation, as measured by decreases in CD38 and HLA-DR expression on CD4+ and CD8+ cells over the course of the study, were observed in both the total patient population (Table 2) and in virologically suppressed patients (Table 2; Figure 2).

The percentage of apoptotic (CD95+) CD4+ cells in suppressed patients significantly increased from baseline to Week 48 (*P* = .0142; Table 2; Figure 2). The percentage of apoptotic CD8+ cells, on the other hand, significantly decreased from baseline to Week 48 in suppressed patients (*P* = .0025; Table 2; Figure 2).

Changes in immune replicative senescence were measured by changes in the frequency of CD4+/CD28− or CD8+/CD28− cells (Table 2). There was little change in the expression of costimulatory marker CD28+ on CD4+ cells from baseline to Week 48 in the total patient population or the virologically suppressed group (Table 2). There was a small decrease in the expression of CD28+ on CD8+ cells in the total patient population and the virologically suppressed population from baseline to Week 48 (Table 2).

### 3.3. Immune Function

**Figure 3:**
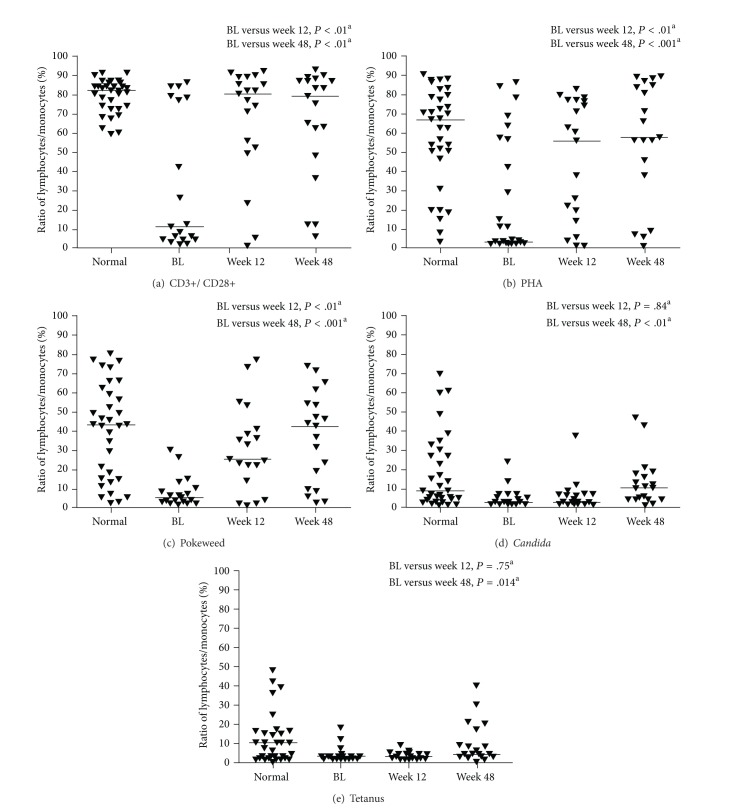
CD4+ lymphocyte proliferation in (a) CD3+/CD28+, (b) phytohemagglutinin, (c) pokeweed, (d) *Candida*, and (e) tetanus. Line represents median; ^a^Wilcoxon rank-sum test; BL: baseline; PHA: phytohemagglutinin.

**Figure 4:**
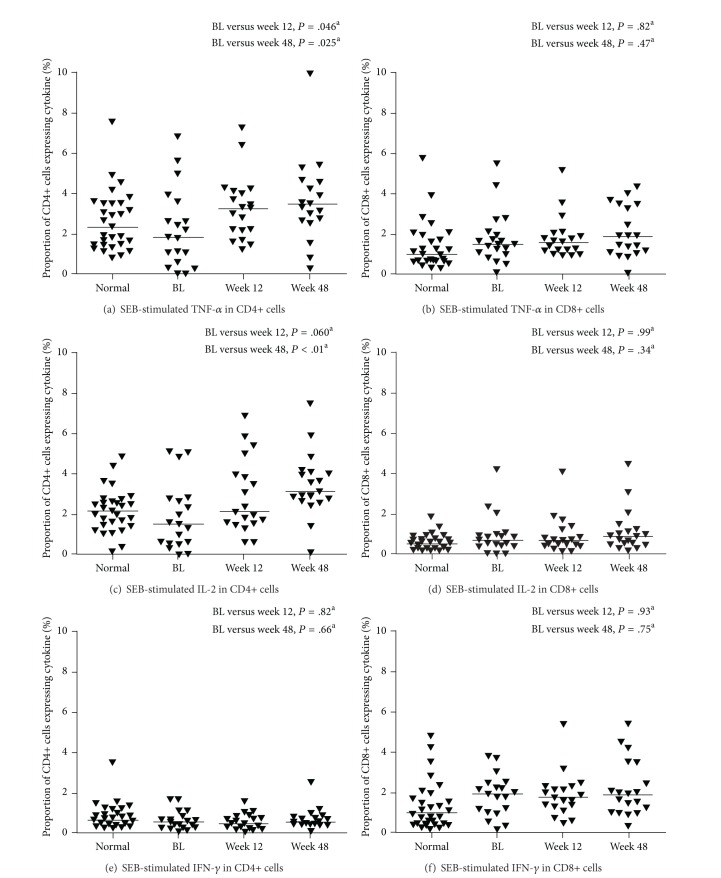
Staphylococcal enterotoxin B-stimulated cytokine expression in virologically suppressed patients. Line represents median; ^a^Wilcoxon rank-sum test; TNF-*α*: tumor necrosis factor-alpha; IL-2: interleukin-2; IFN-*γ*: interferon-gamma; SEB: staphylococcal enterotoxin B; BL: baseline.

The ability of CD4+ lymphocytes to respond to mitogens and recall antigens improved in GRACE patients over the course of the study. Proliferation in response to CD3+/CD28+ and PHA was at, or near, normal levels by Week 12 in virologically suppressed patients, and proliferation in response to pokeweed and *Candida* was at normal levels by Week 48 (Figure 3). Intracellular production of TNF-*α* and IL-2 also increased during the study. Tumor necrosis factor-alpha and IL-2 significantly increased in staphylococcal enterotoxin B-stimulated CD4+ cells of virologically suppressed patients by Week 48; there was no significant change in IFN-*γ* in the stimulated CD4+ cells (Figure 4).

## 4. Discussion 

Few published studies within clinical trials have prospectively assessed in vitro changes in immune function as measured by lymphocyte proliferation [34, 35], and none of these assessed intracellular cytokine production in response to ARV therapy. This substudy from the GRACE trial evaluated T-cell function in a racially diverse, treatment-experienced population comprised of more than 30% women. As expected, based on results from previous studies [24, 27, 28], DRV/r-based therapy resulted in increases in CD4+ cell counts and decreases in CD8+ counts in virologically suppressed patients, with an improved CD4+/CD8+ ratio. In addition, we found that DRV/r-based ARV therapy was associated with progressive functional immune recovery over 48 weeks in virologically suppressed patients, as demonstrated by improved lymphocyte response to mitogens and recall antigens. This suggests that not only do the CD4+ cell counts of HIV-1-infected patients improve during DRV/r-based therapy, but their ability to respond in vitro to immune stimuli may improve as well. Results from this report are consistent with a recent study that demonstrated significant decreases in immune activation and apoptosis in CD4+ and CD8+ T cells and a decrease in immune CD8+ senescence following ARV therapy [36].

While there have been studies evaluating the influence of ARV therapy on immune parameters, data typically represent class-specific rather than regimen-specific treatment approaches. For example, therapy with integrase inhibitors has been shown to result in larger CD4+ cell count increases compared with other ARV classes [29, 37, 38]. The work shown here represents a regimen-specific study, with combination DRV/r treatment in addition to an optimized background regimen that could differ across patients. Unfortunately, we cannot assess differences between regimens, as our study was not comparative.

The reduction in T-cell activation seen here has previously been shown to correlate with the reduction in viral load, as well as an improved response to recall antigens [24]. The observed HIV-1-induced increase in apoptotic CD95+/CD4+ cells has been suggested to be independent of ARV activity; the PI saquinavir has been shown to decrease CD95 expression in CD4+ cells from healthy donors whose cells were previously briefly incubated with HIV-1 virus [25]. Although anti-CD95-induced apoptosis declines with therapy, this change is only occasionally associated with a reduction in expression of CD95 on T lymphocytes [39]. It is thus not surprising that in our study CD95 expression on T cells did not decline. In the GRACE study, no improvement in the CD4+/CD28− or CD8+/CD28− phenotype was seen, consistent with other studies that have suggested that CD28 expression is not normalized in HIV-1–infected patients who have undergone up to 3 years of ARV therapy [40, 41].

One possible limitation to this study is the small sample size, which may limit data interpretation. The study aimed to enroll 50 women and 50 men, but, due to a delayed start date, after initiation of the GRACE study, the patient pool for this substudy was limited and the enrollment goal was not reached. Nonetheless, the data obtained are similar to those of other trials. Both immune phenotype and function of CD4+ and CD8+ cells were significantly improved in treatment-experienced patients receiving DRV/r-based therapy, as evidenced by positive changes in the capacity to proliferate and the expression of intracellular cytokines by CD4+ cells. The functional recovery observed in virologically suppressed patients, as assessed by proliferative response and intracellular cytokine expression, was also seen in nonsuppressed patients, although to a lesser degree.

Antiretroviral therapy has been available for 25 years; however, there has been limited research on immune recovery after long-term treatment. Furthermore, the focus of immune recovery has historically been an assessment of CD4+ cell counts. In the current study, in addition to the evaluation of CD4+ cell counts, CD4+ cell activation (CD38+/HLA-DR+, CD95+), function, and senescence (CD28−) were measured as well as CD8+ cell counts, activation, function, and senescence. It should be noted that, as observed in this trial, despite the improvements in immune phenotype and function seen with ARV therapy, complete normalization of CD4+ and CD8+ parameters is rarely achieved in HIV-1-infected individuals [24, 42]. However, we hypothesize that there may be some clinical benefit even in patients who do not experience measurable or significant increases in CD4+ cell counts. It is feasible that, in patients with higher baseline CD4 levels, measurable increases in CD4+ cell counts may not be observed [43].

Previous studies have shown that despite CD4+ cell count normalization, the pretreatment nadir CD4+ count and level of CD28 CD4+ coexpressing lymphocytes determine ongoing immune competence, including responses to immunization [40]. Despite the successful HIV therapy noted in the GRACE substudy, there seemed to be a higher frequency of the CD8+ CD28− phenotype. This possible treatment-resistant expansion and persistence of cells with a senescence phenotype may have potential for long-term cardiovascular [44], metabolic, and other aging-associated consequences.

This GRACE substudy demonstrates that DRV/r-based therapy improved CD4+ cell recovery and was associated with progressive functional immune recovery over the 48-week study period. Thus, initiation of ARV therapy may be required not only to restore immune function, but also to diminish the effects of chronic inflammation.

## Figures and Tables

**Table 1:** Demographics and baseline characteristics of patients enrolled into the immunology substudy of GRACE.

Parameter		All HIV+ patients (*N* = 32)	Virologically suppressed^a ^patients at Wk 48 (*n* = 19)
Sex, *n* (%)	Men	19 (59.4)	13 (68.4)
	Women	13 (40.6)	6 (31.6)
Race, *n* (%)	Black	15 (46.9)	8 (42.1)
	Hispanic	10 (31.3)	6 (31.6)
	White	7 (21.9)	5 (26.3)
Mean (SD) viral load, log_10_ copies/mL		4.74 (0.84)	4.76 (0.82)
Median (range) CD4+ count, cells/mm^3^		191 (2, 463)	222 (2, 398)

^a^HIV-1 RNA < 50 copies/mL at Wk 48 (or at Wks 36 and 52 if a Wk 48 sample was not available).

**Table 2:** Immune phenotype over the course of the GRACE immunology substudy.

Median (range)	Baseline		Wk 12		Wk 48	
	All^a^	Suppressed^b^	All^a^	Suppressed^b^	All^a^	Suppressed^b^
CD4+ (%)	15 (1, 33)	15 (1, 33)	16 (2, 36)	17 (2, 36)	21 (3, 40)	21 (5, 40)
CD8+ (%)	60.5 (39, 83)	62 (42, 83)	59 (35, 84)	55 (36, 84)	50 (27, 87)	49 (27, 87)
CD4+/CD8+ ratio (%)	0.22 (0.01, 0.70)	0.22 (0.01, 0.70)	0.30 (0.03, 1.00)	0.36 (0.03, 1.00)	0.41 (0.04, 1.21)	0.52 (0.08, 1.21)
CD4+/CD38+/DR+ (%)	22.5 (10, 88)	22 (10, 88)	19 (9, 76)	18 (9, 53)	13 (2, 36)	11 (2, 36)
CD8+/CD38+/DR+ (%)	48.5 (18, 76)	47 (18, 76)	36.5 (11, 71)	34 (11, 62)	25 (4, 68)	22 (4, 52)
CD4+/CD95+ (%)	15 (1, 35)	16 (1, 35)	21 (3, 38)	22 (3, 38)	23 (4, 41)	24 (6, 41)
CD8+/CD95+ (%)	71.5 (40, 87)	70 (40, 87)	69.5 (44, 88)	66 (44, 88)	55 (32, 80)	49 (32, 79)
CD3+/CD4+/CD28+ (%)	93 (46, 99)	95 (56, 99)	94 (67, 100)	94 (70, 100)	96 (67, 99)	96 (67, 99)
CD3+/CD8+/CD28+ (%)	59.5 (39, 86)	62 (39, 85)	50 (39, 79)	50.5 (39, 79)	56 (23, 82)	56 (35, 82)

^a^ *n* = 32 at baseline, *n* = 30 at Wk 12 and *n* = 25 at Wk 48; ^b^HIV-RNA < 50 copies/mL at Wk 48 (or at Wks 36 and 52 if a Wk 48 sample was not available), *n* = 19.

## References

[1] Kestens L, Vanham G, Vereecken C (1994). Selective increase of activation antigens HLA-DR and CD38 on CD4+ CD45RO+ T lymphocytes during HIV-1 infection. *Clinical and Experimental Immunology*.

[2] Levacher M, Hulstaert F, Tallet S, Ullery S, Pocidalo JJ, Bach BA (1992). The significance of activation markers on CD8 lymphocytes in human immunodeficiency syndrome: staging and prognostic value. *Clinical and Experimental Immunology*.

[3] Giorgi JV, Ho H-N, Hirji K (1994). CD8+ lymphocyte activation at human immunodeficiency virus type 1 seroconversion: development of HLA-DR+ CD38- CD8+ cells is associated with subsequent stable CD4+ cell levels. *Journal of Infectious Diseases*.

[4] Klein SA, Dobmeyer JM, Dobmeyer TS (1997). Demonstration of the Th1 to Th2 cytokine shift during the course of HIV-1 infection using cytoplasmic cytokine detection on single cell level by flow cytometry. *AIDS*.

[5] Liu Z, Cumberland WG, Hultin LE, Prince HE, Detels R, Giorgi JV (1997). Elevated CD38 antigen expression on CD8+ T cells Is a stronger marker for the risk of chronic HIV disease progression to AIDS and death in the multicenter AIDS Cohort study than CD4+ cell count, soluble immune activation markers, or combinations of HLA-DR and CD38 expression. *Journal of Acquired Immune Deficiency Syndromes and Human Retrovirology*.

[6] Dion M-L, Poulin J-F, Bordi R (2004). HIV infection rapidly induces and maintains a substantial suppression of thymocyte proliferation. *Immunity*.

[7] Landay A, Ohlsson-Wilhelm B, Giorgi JV (1990). Application of flow cytometry to the study of HIV infection. *AIDS*.

[8] Margolick JB, Munoz A, Donnenberg AD (1995). Failure of T-cell homeostasis preceding AIDS in HIV-1 infection. The Multicenter AIDS Cohort study. *Nature Medicine*.

[9] Kestens L, Vanham G, Gigase P (1992). Expression of activation antigens, HLA-DR and CD38, on CD8 lymphocytes during HIV-1 infection. *AIDS*.

[10] Appay V, Almeida JR, Sauce D, Autran B, Papagno L (2007). Accelerated immune senescence and HIV-1 infection. *Experimental Gerontology*.

[11] Aries SP, Schaaf B, Muller C, Dennin RH, Dalhoff K (1995). Fas (CD95) expression on CD4+ T cells from HIV-infected patients increases with disease progression. *Journal of Molecular Medicine*.

[12] Mogensen TH, Melchjorsen J, Larsen CS, Paludan SR (2010). Innate immune recognition and activation during HIV infection. *Retrovirology*.

[13] Peter ME, Ehret A, Berndt C, Krammer PH (1997). AIDS and the death receptors. *British Medical Bulletin*.

[14] Bentwich Z, Kalinkovich A, Weisman Z, Grossman Z (1998). Immune activation in the context of HIV infection. *Clinical and Experimental Immunology*.

[15] Grossman Z, Meier-Schellersheim M, Paul WE, Picker LJ (2006). Pathogenesis of HIV infection: what the virus spares is as important as what it destroys. *Nature Medicine*.

[16] Hazenberg MD, Hamann D, Schuitemaker H, Miedema F (2000). T cell depletion in HIV-1 infection: how CD4+ T cells go out of stock. *Nature Immunology*.

[17] Trimble LA, Shankar P, Patterson M, Daily JP, Lieberman J (2000). Human immunodeficiency virus-specific circulating CD8 T lymphocytes have down-modulated CD3*ζ* and CD28, key signaling molecules for T-cell activation. *Journal of Virology*.

[18] Vingerhoets JH, Vanham GL, Kestens LL (1995). Increased cytolytic T lymphocyte activity and decreased B7 responsiveness are associated with CD28 down-regulation on CD8+ T cells from HIV-infected subjects. *Clinical and Experimental Immunology*.

[19] Parish ST, Wu JE, Effros RB (2010). Sustained CD28 expression delays multiple features of replicative senescence in human CD8 T lymphocytes. *Journal of Clinical Immunology*.

[20] Avelino-Silva VI, Ho Y-L, Avelino-Silva TJ, Santos SDS (2011). Aging and HIV infection. *Ageing Research Reviews*.

[21] Wood KL, Twigg HL, Doseff AI (2009). Dysregulation of CD8+ lymphocyte apoptosis, chronic disease, and immune regulation. *Frontiers in Bioscience*.

[22] Giorgi JV, Liu Z, Hultin LE, Cumberland WG, Hennessey K, Detels R (1993). Elevated levels of CD38+CD8+ T cells in HIV infection add to the prognostic value of low CD4+ T cell levels: results of 6 years of follow-up. *Journal of Acquired Immune Deficiency Syndromes*.

[23] Clerici M, Shearer GM (1993). A T_H_1 → T_H_2 switch is a critical step in the etiology of HIV infection. *Immunology Today*.

[24] Autran B, Carcelain G, Li TS (1997). Positive effects of combined antiretroviral therapy on CD4+ T cell homeostasis and function in advanced HIV disease. *Science*.

[25] Estaquier J, Leliévre J-D, Petit F (2002). Effects of antiretroviral drugs on human immunodeficiency virus type 1-induced CD4+ T-cell death. *Journal of Virology*.

[26] Badley AD, Parato K, Cameron DW (1999). Dynamic correlation of apoptosis and immune activation during treatment of HIV infection. *Cell Death and Differentiation*.

[27] Goicoechea M, Smith DM, Liu L (2006). Determinants of CD4+ T cell recovery during suppressive antiretroviral therapy: association of immune activation, T cell maturation markers, and cellular HIV-1 DNA. *Journal of Infectious Diseases*.

[28] Riddler SA, Haubrich R, DiRienzo AG (2008). Class-sparing regimens for initial treatment of HIV-1 infection. *The New England Journal of Medicine*.

[29] Pichenot M, Deuffic-Burban S, Cuzin L, Yazdanpanah Y (2012). Efficacy of new antiretroviral drugs in treatment-experienced HIV-infected patients: a systematic review and meta-analysis of recent randomized controlled trials. *HIV Medicine*.

[30] Llibre JM, Buzón MJ, Massanella M (2012). Treatment intensification with raltegravir in subjects with sustained HIV-1 viraemia suppression: a randomized 48-week study. *Antiviral Therapy*.

[31] Hatano H, Hayes TL, Dahl V (2011). A randomized, controlled trial of raltegravir intensification in antiretroviral-treated, HIV-infected patients with a suboptimal CD4+ T cell response. *Journal of Infectious Diseases*.

[32] Byakwaga H, Kelly M, Purcell DFJ (2011). Intensification of antiretroviral therapy with raltegravir or addition of hyperimmune bovine colostrum in HIV-infected patients with suboptimal CD4+ T-cell response: a randomized controlled trial. *Journal of Infectious Diseases*.

[33] Currier J, Bridge DA, Hagins D (2010). Sex-based outcomes of Darunavir-Ritonavir therapy: a single-group trial. *Annals of Internal Medicine*.

[34] Angel JB, Parato KG, Kumar A (2001). Progressive human immunodeficiency virus-specific immune recovery with prolonged viral suppression. *Journal of Infectious Diseases*.

[35] Ullum H, Katzenstein T, Aladdin H (1999). Immunological changes in Human Immunodeficiency Virus (HIV)-infected individuals during HIV-specific protease inhibitor treatment. *Scandinavian Journal of Immunology*.

[36] Kaplan RC, Parrinello CM, Hodis HN Impact of HAART initiation on immune regulation (activation/senescence) in aging HIV-infected women: Women's Interagency HIV study.

[37] Garrido C, Rallón N, Soriano V (2012). Mechanisms involved in CD4 gains in HIV-infected patients switched to raltegravir. *AIDS*.

[38] Steigbigel RT, Cooper DA, Kumar PN (2008). Raltegravir with optimized background therapy for resistant HIV-1 infection. *The New England Journal of Medicine*.

[39] Gougeon M-L, Lecoeur H, Sasaki Y (1999). Apoptosis and the CD95 system in HIV disease: impact of Highly Active Anti-Retroviral Therapy (HAART). *Immunology Letters*.

[40] Lange CG, Lederman MM, Medvik K (2003). Nadir CD4+ T-cell count and numbers of CD28+ CD4+ T-cells predict functional responses to immunizations in chronic HIV-1 infection. *AIDS*.

[41] Niehues T, Horneff G, Knipp S, Adams O, Wahn V (2000). Treatment-resistant expansion of CD8+CD28-cells in pediatric HIV infection. *Pediatric Research*.

[42] Lange CG, Lederman MM (2003). Immune reconstitution with antiretroviral therapies in chronic HIV-1 infection. *Journal of Antimicrobial Chemotherapy*.

[43] Hill A, Montaner J, Lederman M, Cutrell A, Tortell S, Thorborn D Discordant CD4/RNA responses to HAART are strongly associated with high baseline CD4 count and low HIV RNA: analysis of 406 naive patients.

[44] Kaplan RC, Sinclair E, Landay AL (2011). T cell activation and senescence predict subclinical carotid artery disease in HIV-infected women. *Journal of Infectious Diseases*.

